# Interferon inducible GBPs restrict *Burkholderia thailandensis* motility induced cell-cell fusion

**DOI:** 10.1371/journal.ppat.1008364

**Published:** 2020-03-09

**Authors:** David E. Place, Benoit Briard, Parimal Samir, Rajendra Karki, Anannya Bhattacharya, Clifford S. Guy, Jennifer L. Peters, Sharon Frase, Peter Vogel, Geoffrey Neale, Masahiro Yamamoto, Thirumala-Devi Kanneganti

**Affiliations:** 1 Department of Immunology, St. Jude Children’s Research Hospital, Memphis, Tennessee, United States of America; 2 Cell and Tissue Imaging Center, St. Jude Children’s Research Hospital, Memphis, Tennessee, United States of America; 3 Veterinary Pathology Core, St. Jude Children’s Research Hospital, Memphis, Tennessee, United States of America; 4 Hartwell Center for Bioinformatics & Biotechnology, St. Jude Children’s Research Hospital, Memphis, Tennessee, United States of America; 5 Department of Immunoparasitology, Osaka University, 3–1 Yamadaoka, Suita, Osaka, Japan; University of Toronto, CANADA

## Abstract

Innate immunity responds to pathogens by producing alarm signals and activating pathways that make host cells inhospitable for pathogen replication. The intracellular bacterium *Burkholderia thailandensis* invades the cytosol, hijacks host actin, and induces cell fusion to spread to adjacent cells, forming multinucleated giant cells (MNGCs) which promote bacterial replication. We show that type I interferon (IFN) restricts macrophage MNGC formation during *B*. *thailandensis* infection. Guanylate-binding proteins (GBPs) expressed downstream of type I IFN were required to restrict MNGC formation through inhibition of bacterial Arp2/3-dependent actin motility during infection. GTPase activity and the CAAX prenylation domain were required for GBP2 recruitment to *B*. *thailandensis*, which restricted bacterial actin polymerization required for MNGC formation. Consistent with the effects in in vitro macrophages, *Gbp*2^−/−^, *Gbp*5^−/−^, *Gbp*^Chr3^-KO mice were more susceptible to intranasal infection with *B*. *thailandensis* than wildtype mice. Our findings reveal that IFN and GBPs play a critical role in restricting cell-cell fusion and bacteria-induced pathology during infection.

## Author summary

The intracellular bacterium *Burkholderia thailandensis* and its relatives *B*. *pseudomallei* and *B*. *mallei* each invade host cells and hijack the actin cytoskeleton polymerization machinery to transmit to neighboring cells by cell-cell fusion, a transmission strategy that is unique to this family. The high antibiotic resistance of the *Burkholderia* family underscores the need to understand how the immune system can control infections. Here, we show that the interferon immune response upregulates a family of immune proteins, the guanylate binding proteins (GBPs), to counter the bacterial intracellular motility and, as a consequence, cell-cell fusion. Infected macrophages extensively fuse when lacking key molecules in this immune pathway, and mice lacking the GBP2 or GBP5 proteins are 100-1000-fold more susceptible to infection than wildtype mice, highlighting the critical role this immune pathway plays in restricting bacterial infection and cell-cell fusion. We also found that mice lacking GBPs were protected if bacteria lacked a critical virulence factor, VgrG5, that is required for cell-cell fusion, highlighting that cell-cell fusion is dependent on both bacteria-mediated cytoskeleton remodeling as well as VgrG5. Together, this study provides insight into an intricate host-pathogen interaction key to the virulence of the *Burkholderia* family.

## Introduction

Interferon (IFN) signaling pathways are critical regulators of host immunity to many viral and bacterial infectious diseases, leading to the expression of a number of IFN-stimulated genes (ISGs) with antiviral, antibacterial, or pathogenic activities [1,2]. These ISGs include the dynamin-like GTPase myxovirus resistance 1 (Mx1), the IFN-inducible transmembrane (IFITM) proteins, the tripartite-motif family (TRIM) proteins, and the guanylate-binding proteins (GBPs) that are highly upregulated in response to IFN signaling and are important for restricting intracellular infections [2–4]. While these responses are important for restricting many microbes in a cell-autonomous manner, many pathogens are able to overcome these defenses. *Burkholderia thailandensis* is an opportunistic intracellular pathogen that induces multinucleated giant cell (MNGC) formation in macrophage cell lines and is utilized as a model organism for the related and highly virulent *B*. *pseudomallei* and *B*. *mallei*, which are considered potential bioweapons [5–7]. Previous studies have also shown that *Burkholderia* infections are critically controlled by multiple inflammasomes, pyroptotic cell death, and the pyroptosis-regulated inflammatory cytokine IL-18 [8–10]. *Burkholderia* infection-induced pathology is also mediated by excessive release of pyroptosis-regulated IL-1β. The balance of the beneficial and harmful consequences of inflammasome activation and how *Burkholderia*-mediated MNGC formation regulates inflammasome activation has not been well studied.

To drive membrane fusion between cells, *B*. *thailandensis* rapidly escapes from the endosomal compartment and hijacks the host Arp2/3-dependent actin polymerization machinery to drive fusion with neighboring cells [11]. Many pathogens take advantage of the host machinery to induce membrane fusion, facilitating their intracellular or intercellular spread during infection; therefore, understanding how the host immune response counteracts these processes is critical. Because coordinated actin cytoskeleton remodeling is required for cell-cell fusion in multiple cell types and human GBP1 has been shown to regulate actin polymerization during infection by Kaposi’s sarcoma-associated herpesvirus (KSHV) and *Shigella flexneri* [12–18], we hypothesized that cell-cell fusion during *B*. *thailandensis* infection, a process requiring bacteria-mediated actin polymerization, could be restricted by the IFN signaling pathway through expression of GBPs [12,13,18].

In this study we find that GBPs regulated by the type I IFN pathway are required to restrict the formation of MNGCs during *B*. *thailandensis* infection. GBPs restrict *B*. *thailandensis* intercellular spread through the inhibition of bacterial actin-based motility, which is required for cell fusion. Mechanistically, the inhibitory activity of GBP2 required the GTPase activity and CAAX (C representing cysteine, AA two aliphatic residues, and X any C-terminal amino acid) membrane localization domain. Loss of either GBP2 or GBP5 alone was sufficient to render bone marrow-derived macrophages (BMDMs) and mice highly susceptible to infection by *B*. *thailandensis*. The increased susceptibility of GBP-deficient BMDMs and mice also required the bacterial virulence factor VgrG5, which is required for MNGC fusion. Together, these data show that GBPs are potent cell-autonomous proteins that restrict the motility and spread of intracellular *B*. *thailandensis* to neighboring cells by MNGC formation through their ability to regulate actin cytoskeleton dynamics.

## Results

### Type I IFN restricts MNGC formation during *B*. *thailandensis* infection

**Fig 1:**
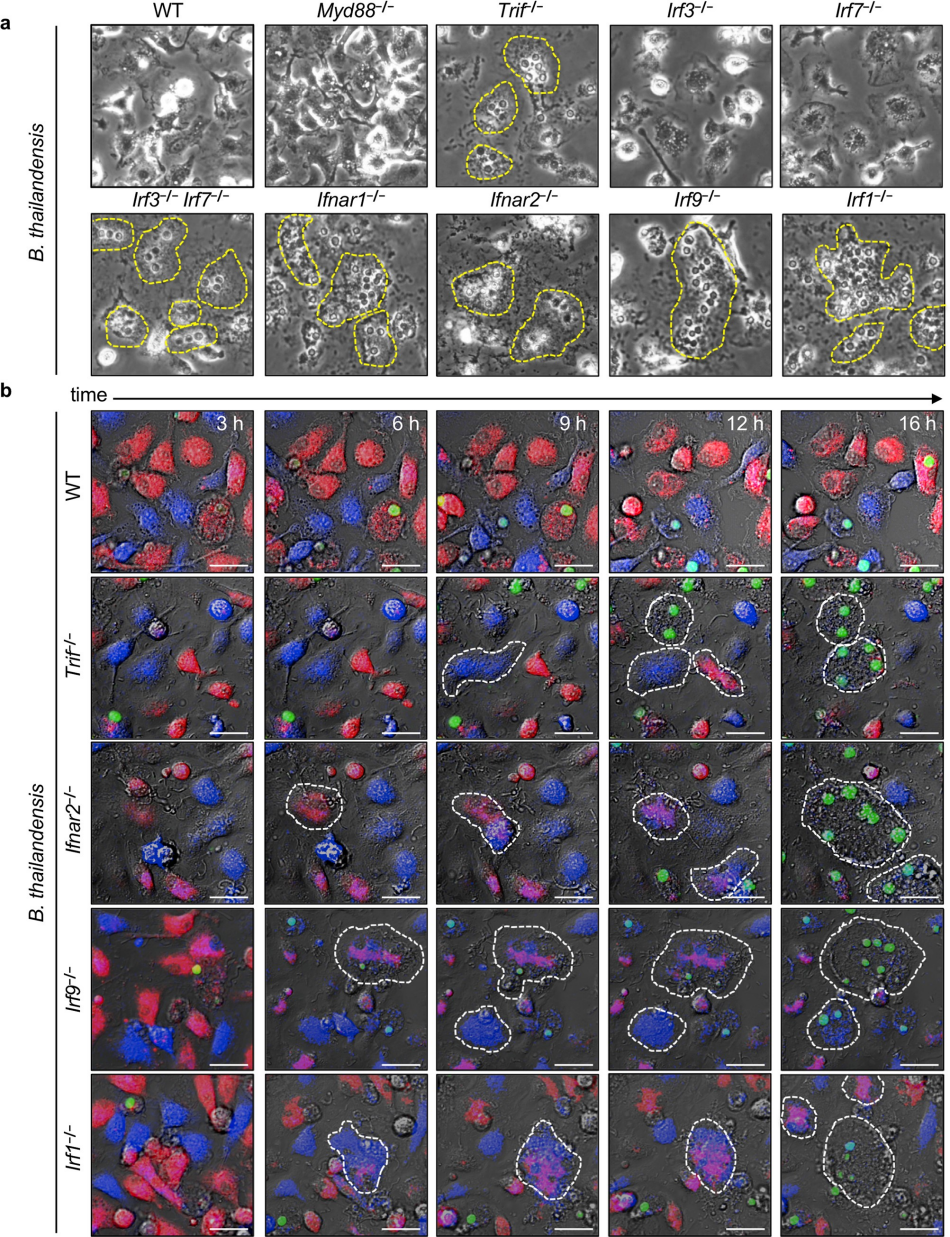
Type I IFN restricts MNGC formation during *B*. *thailandensis* infection. Unprimed BMDMs were infected with *B*. *thailandensis* (MOI 5) and **(a)** phase contrast images (20x) were collected at 20 h post-infection or **(b)** live-microscopy images over time were collected from 1:1 (CellTrace Far Red/CellTrace Violet-labelled) mixed BMDMs of the indicated genotype with Sytox Green staining nuclei of permeabilized cells. Dotted yellow lines indicate boundaries of multinucleated cells in each field. White scale bars indicate 20 μm. Images are representative of at least three independent experiments with live-microscopy images ranging from 3 to 16 h post-infection.

The role of type I IFNs in the immune response to *B*. *thailandensis* infections is poorly characterized. We first determined whether the type I IFN pathway was required to control *B*. *thailandensis* infection in unprimed BMDMs. Upon *B*. *thailandensis* infection, unprimed BMDMs lacking molecules involved in the expression of type I IFNs (TRIF, IRF3/IRF7), the type I IFN receptors (IFNAR1, IFNAR2), or downstream type I IFN signaling regulators (IRF9 and IRF1) clustered in large areas with tightly packed nuclei (**Fig 1A, dashed yellow outline**). This dense aggregation of nuclei suggested IFN signaling-deficient BMDMs underwent cell-cell fusion prior to lysis (**Fig 1A**). BMDMs deficient in MyD88, IRF3, or IRF7 alone, however, did not undergo increased cell-cell fusion (**Fig 1A**). To confirm and visualize the cell-cell fusion dynamics, we labeled BMDMs with two different cytosolic fluorescent dyes (CellTrace Far Red or CellTrace Violet) and utilized live cell fluorescence microscopy to monitor for cytoplasmic mixing of these dyes, indicating a fusion event. We observed that BMDMs lacking TRIF, IFNAR2, IRF9, and IRF1 underwent increased cell fusion (6–12 h), and this fusion preceded cell lysis and uptake of Sytox Green (12–16 h) (**Fig 1B, S4B Fig**). We also found that deletion of signaling molecules that act upstream of type I IFN production including multiple TLRs (2, 3, 4, 5), RIPK2, cGAS/STING, or MAVS pathways, did not increase BMDM fusion during infection while control BMDMs lacking MyD88 and TRIF or IFNAR1 extensively fused (**S1 Fig**). Together these data suggest the critical downstream signaling molecules of type I IFN are important for regulating factors that restrict MNGC formation during *B*. *thailandensis* infection.

### IFN-inducible GBPs are required to restrict *B*. *thailandensis* cell-cell fusion

**Fig 2:**
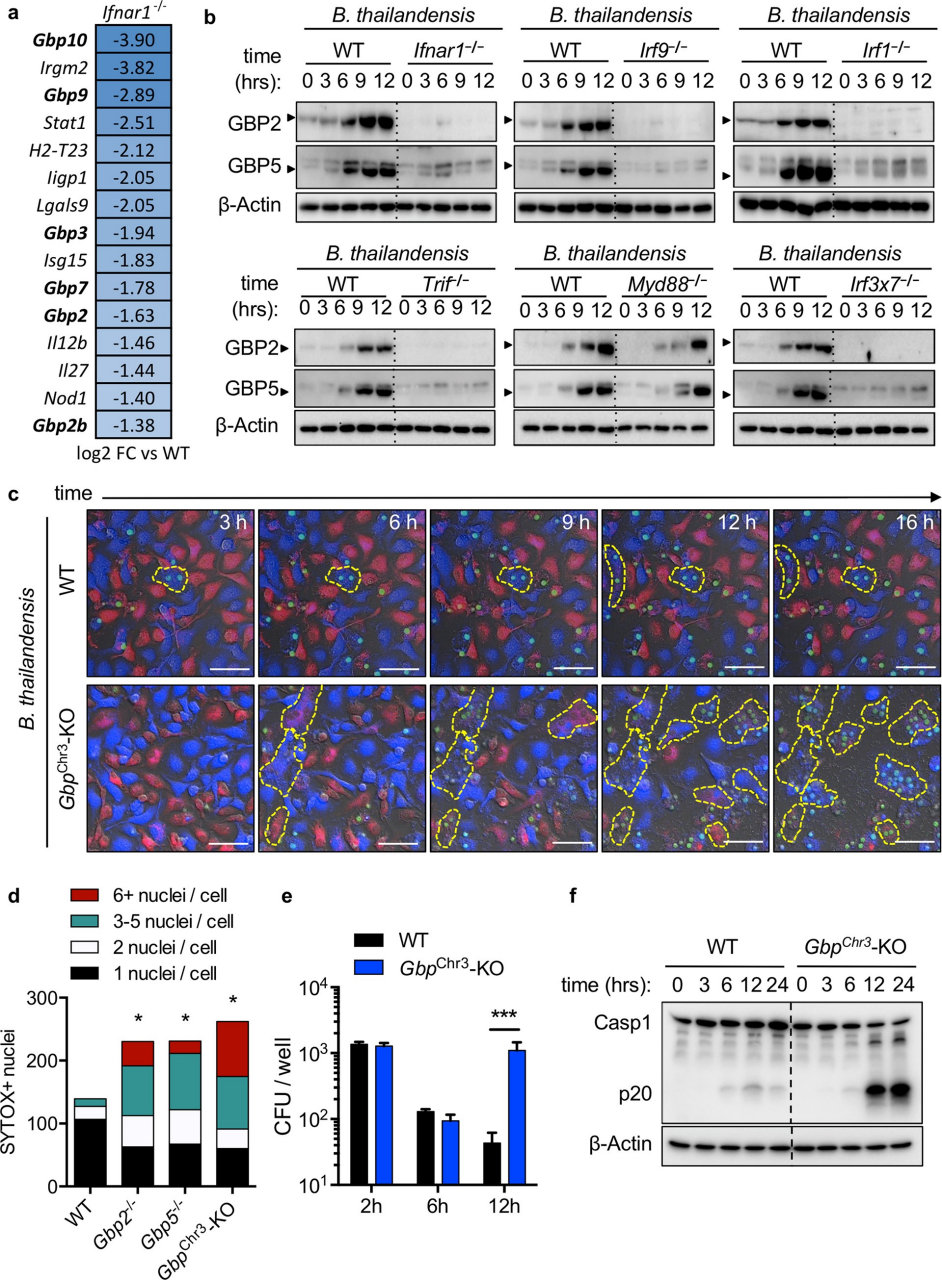
Interferon-inducible GBPs are required to restrict *B*. *thailandensis* cell-cell fusion. Unprimed BMDMs were infected with *B*. *thailandensis* (MOI 5) and **(a)** RNA was isolated for microarray analysis, focusing on genes involved in bacterial immune responses, **(b)** lysed in RIPA buffer for immunoblots at the indicated time-points to assess expression of GBP2 and GBP5 in *Ifnar1*^**−/−**^, *Irf9*^**−/−**^, *Irf1*^**−/−**^, *Trif*^**−/−**^, *Myd88*^**−/−**^, and *Irf3*^**−/−**^*Irf7*^**−/−**^ (*Irf3x7*^**−/−**^) BMDMs. **(c)** Live confocal microscopy of fluorescently labeled BMDMs with Sytox Green to visualize cell-cell fusion and death. **(d)** The extent of multinucleation was measured by counting the nuclei present in each observed multinucleated cell in over three images per genotype from live microscopy images. **(e)** Intracellular bacterial colony forming units (CFU) were determined at indicated time-points post-infection. **(f)** Caspase-1 cleavage following infection with *B*. *thailandensis* at indicated time-points in wildtype and *Gbp*^Chr3^-KO BMDMs. White scale bars indicate 20 μm. Significant differences were determined by one-way ANOVA with Dunnett’s multiple comparison test with *P < 0.05, **P < 0.01, ***P < 0.001 **(d, e)**. Data are representative of **(a)** one repeat from two independent samples per condition or **(b-f)** three or more independent experiments. Refers to Supplementary Videos 1 and 2.

To determine which ISGs confer resistance against cell fusion in macrophages, we performed microarray analysis on wildtype and *Ifnar1*^**−/−**^ BMDMs infected with *B*. *thailandensis*. Expression of multiple GBPs was decreased in *Ifnar1*^**−/−**^ macrophages compared with wildtype cells (**Fig 2A**). Consistent with the microarray, protein levels of GBP2 and GBP5 were reduced in the *Ifnar1*^**−/−**^, *Irf9*^**−/−**^, *Irf1*^**−/−**^, *Trif*^**−/−**^, and *Irf3*^**−/−**^*Irf7*^**−/−**^ macrophages that undergo extensive cell-cell fusion during infection but not in *Myd88*^**−/−**^ (**Fig 2B**). To determine whether GBPs were involved in limiting cell fusion during *B*. *thailandensis* infection, we used live cell fluorescence microscopy and found that *Gbp*^Chr3^-KO BMDMs (lacking GBP2, GBP2b (GBP1), GBP3, GBP5, and GBP7) underwent increased cell fusion compared with wildtype BMDMs (**Fig 2C, S1 and S2 Videos**). We further quantified the fusion in *Gbp2*^**−/−**^, *Gbp5*^**−/−**^, and *Gbp*^Chr3^-KO BMDMs and found that all GBP-deficient BMDMs formed significantly more multinucleated cells than wildtype BMDMs did (**Fig 2D**). To determine whether intracellular bacterial numbers were similar between wildtype and *Gbp*^Chr3^-KO BMDMs prior to cell fusion or at the start of observable MNGC formation, we quantified viable intracellular bacterial colony forming units (CFUs) at 2 or 6 h post-infection, respectively, and observed no differences compared to wildtype cells (**Fig 2E**). Because MNGC formation allows for an increase in the number of infected cells, we measured intracellular bacterial numbers at 12 h post-infection and observed that the increased cell fusion in *Gbp*^Chr3^-KO BMDMs led to an increase in intracellular bacterial replication (**Fig 2E**). We tested whether IRGB10, another ISG important for control of intracellular *Francisella* [19], was important for restricting *B*. *thailandensis* MNGC formation and inflammasome activation and found no role for IRGB10 (**S2A and S2B Fig**). Except for the presence of multinucleated macrophages and mixing of cell cytoplasm, no apparent changes to the cellular ultrastructure were observed in infected *Gbp*^Chr3^-KO macrophages **(S3 Fig**). Because previous studies have shown GBPs are required for inflammasome-mediated clearance of intracellular bacteria such as *F*. *novicida*, we also investigated whether GBPs were required for inflammasome activation during *B*. *thailandensis* infection by measuring caspase-1 cleavage in unprimed BMDMs over time [20,21]. We found that caspase-1 activation was in fact increased in *Gbp*^Chr3^-KO compared with wildtype BMDMs at multiple time-points following infection, suggesting GBPs are not necessary for *B*. *thailandensis* inflammasome activation in unprimed BMDMs **(Fig 2F).** Increased cell-cell fusion and caspase-1 activation led to an increase in the cell death, as measured by BMDM uptake of Sytox Green during the course of infection in GBP and IFN signaling knockouts (**S4A and S4B Fig**). Together, these findings suggest that the increase in caspase-1 cleavage is a consequence of the increased cell-cell fusion that permits prolonged *B*. *thailandensis* intracellular replication in MNGCs. Priming of GBP-deficient BMDMs with IFNβ and IFNγ did not protect from *B*. *thailandensis*-induced cell-cell fusion or cell death, but IFNγ protected *Ifnar1*^−/−^ BMDMs **(S4C–S4E Fig).** Overall, these results demonstrate that GBPs are required for restricting *B*. *thailandensis*-induced cell fusion and intercellular spread.

### GBPs inhibit Arp2/3-dependent actin motility required for MNGC formation

**Fig 3:**
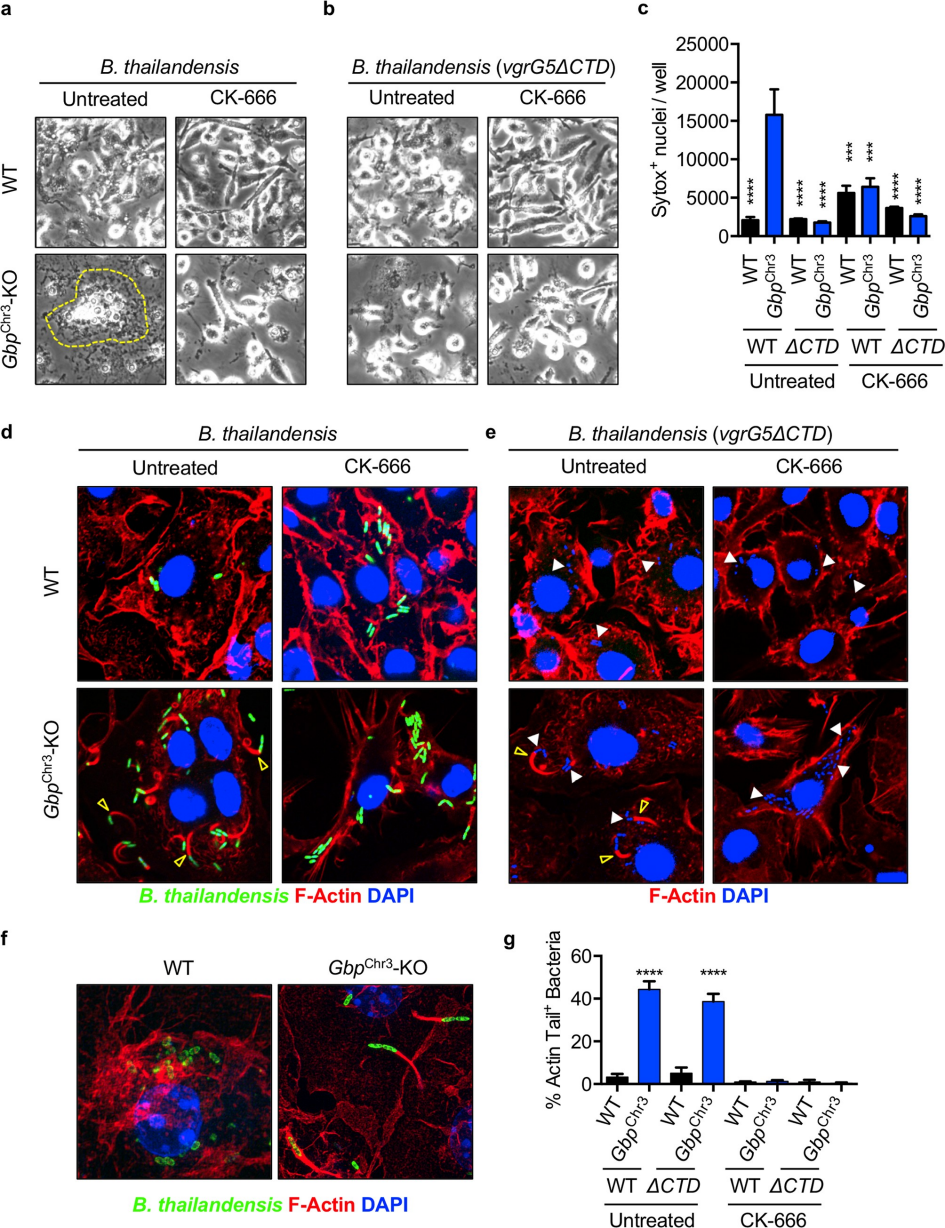
GBPs inhibit Arp2/3-dependent actin motility required for MNGC formation. Unprimed BMDMs were infected (MOI 5) with *B*. *thailandensis* (GFP^+^) or the fusion-defective mutant as described and after the final wash, CK-666 (200 μM) was added where indicated. Phase-contrast images were collected at 20 h post-infection with **(a)** wildtype *B*. *thailandensis* or **(b)** *B*. *thailandensis vgrG5 ΔCTD*, with or without CK-666. Dotted yellow lines indicate multinucleated cells. **(c)** Uptake of Sytox Green at 20 h post-infection in CK-666 treated BMDMs. Fluorescent microscopy images of BMDMs infected with **(d)** wildtype *B*. *thailandensis* or **(e)** *B*. *thailandensis vgrG5ΔCTD*, with or without CK-666 were collected at 6 h post-infection. **(f)** Super-resolution microscopy images were collected from 6 h samples. **(g)** The percentage (%) actin tail-positive wildtype bacteria (indicated by empty yellow arrow in **(d)** and **(e)**) or *B*. *thailandensis vgrG5ΔCTD* (stained with DAPI, indicated by white arrows) were quantified from at least three fields per condition normalized to total bacterial counts. Significance was determined by one-way ANOVA with the Holm-Sidak multiple comparisons test, ****P < 0.00001, ***P < 0.0001 (**c, g**). Images and quantification are representative of **(a,b,c)** at least three independent repeats, **(d,e,f)** one representative of three experiments, or **(g)** quantified from at least three fields from one independent experiment.

The precise host-pathogen mechanisms for MNGC formation during *B*. *thailandensis* infection are not completely understood, although the *B*. *pseudomallei* group (consisting of *B*. *pseudomallei*, *B*. *mallei*, and *B*. *thailandensis*) virulence protein BimA is known to hijack the host Arp2/3 actin polymerization machinery to polymerize F-actin and facilitate bacterial cell-cell spreading [11,22–24]. In cooperation with F-actin polymerization, the bacterial factor VgrG5 is also required for *B*. *thailandensis* MNGC formation [6,7]. Specifically, the C-terminal domain (CTD) of VgrG5 is required to induce cell fusion [6,7]. To determine whether VgrG5 or host actin remodeling are necessary for the increased fusion in *Gbp*^Chr3^-KO BMDMs, or whether loss of GBPs alone is sufficient to increase fusion, we infected wildtype and *Gbp*^Chr3^-KO BMDMs with *B*. *thailandensis* or *B*. *thailandensis vgrG5ΔCTD* for 2 h, followed by treatment with the Arp2/3-specific inhibitor CK-666 [25]. Inhibition of Arp2/3 (CK-666 treatment) restricted increased cell fusion in *Gbp*^Chr3^-KO BMDMs, highlighting the critical role of Arp2/3-mediated actin polymerization in MNGC formation (**Fig 3A**). To dissect whether loss of GBPs alone may render BMDMs more likely to fuse in the absence of the *B*. *thailandensis* VgrG5 activity, we infected BMDMs and observed no increase in MNGC formation in *Gbp*^Chr3^-KO BMDMs (**Fig 3B**). Inhibition of Arp2/3-mediated actin polymerization also limited the increased cell-cell fusion mediated increase in cell death in *Gbp*^Chr3^-KO BMDMs (**Fig 3C**). Because we observed a role for both host cell Arp2/3-mediated actin polymerization and the bacterial effector VgrG5, we examined bacteria-mediated actin tail formation during infection with *B*. *thailandensis* or *B*. *thailandensis vgrG5ΔCTD*. Infected wildtype BMDMs contained significantly fewer bacteria with characteristic F-actin tails compared with *Gbp*^Chr3^-KO BMDMs, suggesting that GBPs inhibit bacterial F-actin polymerization (**Fig 3D and 3G, yellow arrow**). Furthermore, inhibition of Arp2/3 (CK-666) prevents cell fusion and F-actin tail formation in *Gbp*^Chr3^-KO BMDMs, confirming the critical role of Arp2/3-mediated F-actin tail formation in MNGC formation (**Fig 3D and 3G**). Notably, the CTD of VgrG5 is required for cell fusion but not actin tail formation [6,7], confirming that both actin remodeling and VgrG5 activity are required to drive cell fusion during infection with *B*. *thailandensis* in *Gbp*^Chr3^-KO BMDMs (**Fig 3E and 3G).** We also showed that actin polymerization and membrane protrusion by *B*. *thailandensis* is restricted in wildtype but not *Gbp*^Chr3^-KO BMDMs using super-resolution microscopy (**Fig 3F**). Consistent with previous studies showing the involvement of actin polymerization in mediating fusion between cells [11,16,18], our data suggest that during *B*. *thailandensis* infection, GBPs restrict cell fusion via inhibition of Arp2/3-mediated actin remodeling.

### GBP2 activity requires GTPase and CAAX domains to restrict *B*. *thailandensis*

**Fig 4:**
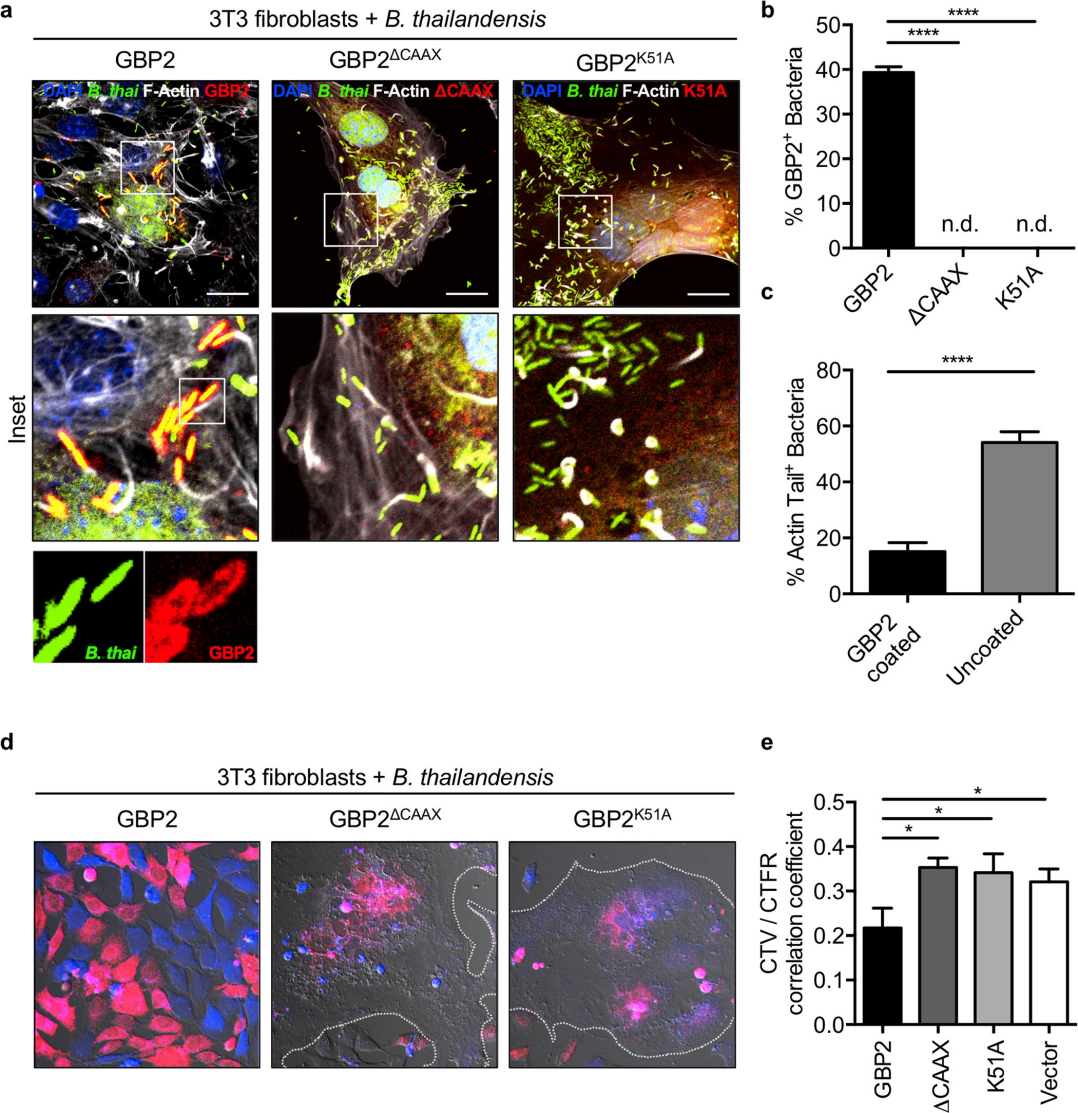
GBP2 activity requires GTPase and CAAX domains to restrict *B*. *thailandensis*. NIH3T3 fibroblasts stably transduced with mCherry-tagged GBP2 (wildtype and mutants, red) and unprimed cells were infected with *B*. *thailandensis* (GFP^+^). (**a-c**) Cells were fixed at 12 h post-infection, stained for F-actin (white), and images were collected for GBP2 localization and F-actin tail quantification. NIH3T3 fibroblasts pre-stained with CellTrace Far Red or CellTrace Violet and seeded overnight at a 1:1 ratio (**d**) were fixed at 24 h post-infection and imaged for cell fusion and (**e**) had fusion quantified by colocalization of the two CellTrace signals. Dotted white line outlines the boundary of large MNGC formed during infection. Images are representative of at least ten images collected for quantification. White scale bars indicate 20 μm. Significance was determined by one-way ANOVA with the Holm-Sidak multiple comparison test, *P < 0.05, ****P < 0.0001 (**b, c, e**).

Human GBP1 inhibits actin polymerization in an oligomerization- and GTPase activity-dependent manner [14,26]. Further, human GBP1 was shown to localize to intracellular *S*. *flexneri* and restrict bacteria-mediated actin polymerization dependent upon its GTPase activity and prenylation domain [15,17,27]. A triple-arginine motif upstream of the CAAX domain of human GBP1 was required to inhibit actin tail formation in *Shigella* infected cells [15]. Unlike human GBP1, however, murine GBP1, GBP2, and GBP5 lack this triple-arginine motif (**S5 Fig, red box**) and murine GBP2 and GBP5 are both required to restrict *B*. *thailandensis*-induced cell fusion (**Fig 2**). Because of these differences between murine and human GBPs, we examined whether two other functional domains in the GBP proteins were required for recruitment to *B*. *thailandensis* and actin regulation. The Ras and Rho family GTPases contain GTPase and CAAX domains which regulate their localization and function, with accessory proteins further regulating their activity and ability to remodel the actin cytoskeleton [28,29]. The IFN-inducible GTPases that possess a C-terminal CAAX domain (GBP1 (GBP2b), GBP2, GBP5) have high homology with the Ras and Rho family GTPases (**S5 Fig)**, suggesting a conserved function in regulating their localization and the actin cytoskeleton. To mechanistically determine how murine GBPs restrict bacteria-mediated actin remodeling, we examined whether murine GBP2 localizes to *B*. *thailandensis* and inhibits the polymerization of F-actin in fibroblasts transduced to express mCherry-tagged GBP2, GBP2^K51A^ (a GTPase activity-deficient mutant), or GBP2^ΔCAAX^ (required for prenylation and membrane localization). We observed that both the CAAX domain, required for prenylation, and the GTPase activity of GBP2 were required for localization to *B*. *thailandensis* (**Fig 4A and 4B**). Recruitment of GBP2 to bacteria restricted the formation of actin tails required for intracellular motility and fusion with neighboring cells (**Fig 4C**). To confirm that the reduction of actin tail formation restricted cell-cell fusion, each of the stably transduced fibroblasts expressing vector control, GBP2, GBP2^ΔCAAX^, or GBP2^K51A^ were stained with CellTrace Far Red or CellTrace Violet, seeded, and infected with *B*. *thailandensis*. After 24 h of infection, we observed that fibroblasts overexpressing GBP2 but not empty vector, GBP2^ΔCAAX^, or GBP2^K51A^ were able to restrict bacteria-induced fusion (**Fig 4D and 4E**). Together, these data show that GBP2 localized to bacteria restricts bacterial actin-based motility dependent upon its GTPase activity and C-terminal CAAX prenylation domain, ultimately restricting bacteria-mediated cell-cell fusion.

### GBPs are required to control *B*. *thailandensis* lung infection

**Fig 5:**
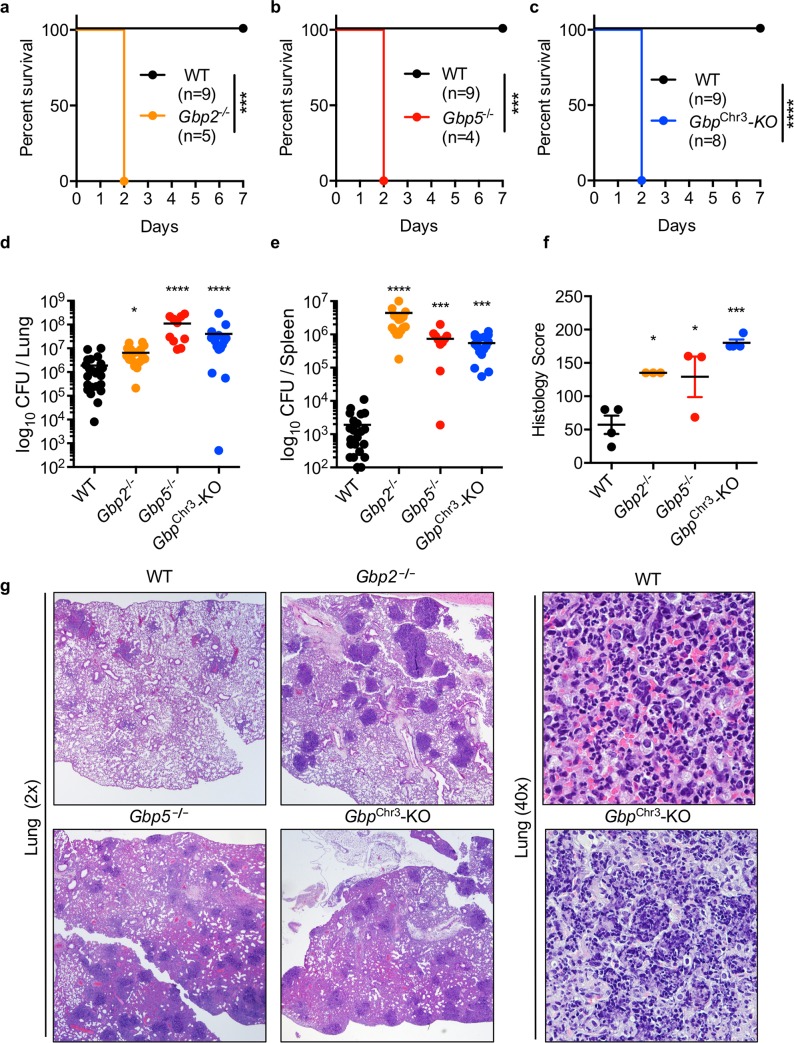
GBPs are required to control *B*. *thailandensis* lung infection. Mice were inoculated intranasally with *B*. *thailandensis* (5 x 10^3^) and **(a-c)** monitored for survival in the indicated knockout mice. Bacterial colony-forming units (CFUs) were determined by serially diluting and plating **(d)** lung and **(e)** spleen homogenates at day 2 post-infection. **(f, g)** Combined semi-quantitative histological pathology scores (described in the Methods) and representative images of stained lung sections (n = 3–4) were generated by a trained pathologist (PV). Wildtype mice in **(a-c)** are the same group and same experiment as knockouts, separated for clarity. Statistical significance was determined by the log-rank test (**a-c**) or one-way ANOVA (**d-f**), *P < 0.05, ***P < 0.001, ****P < 0.0001. Data are representative of **(a-c, f-g)** at least three independent experiments or **(d-e)** pooled from three independent experiments.

**Fig 6:**
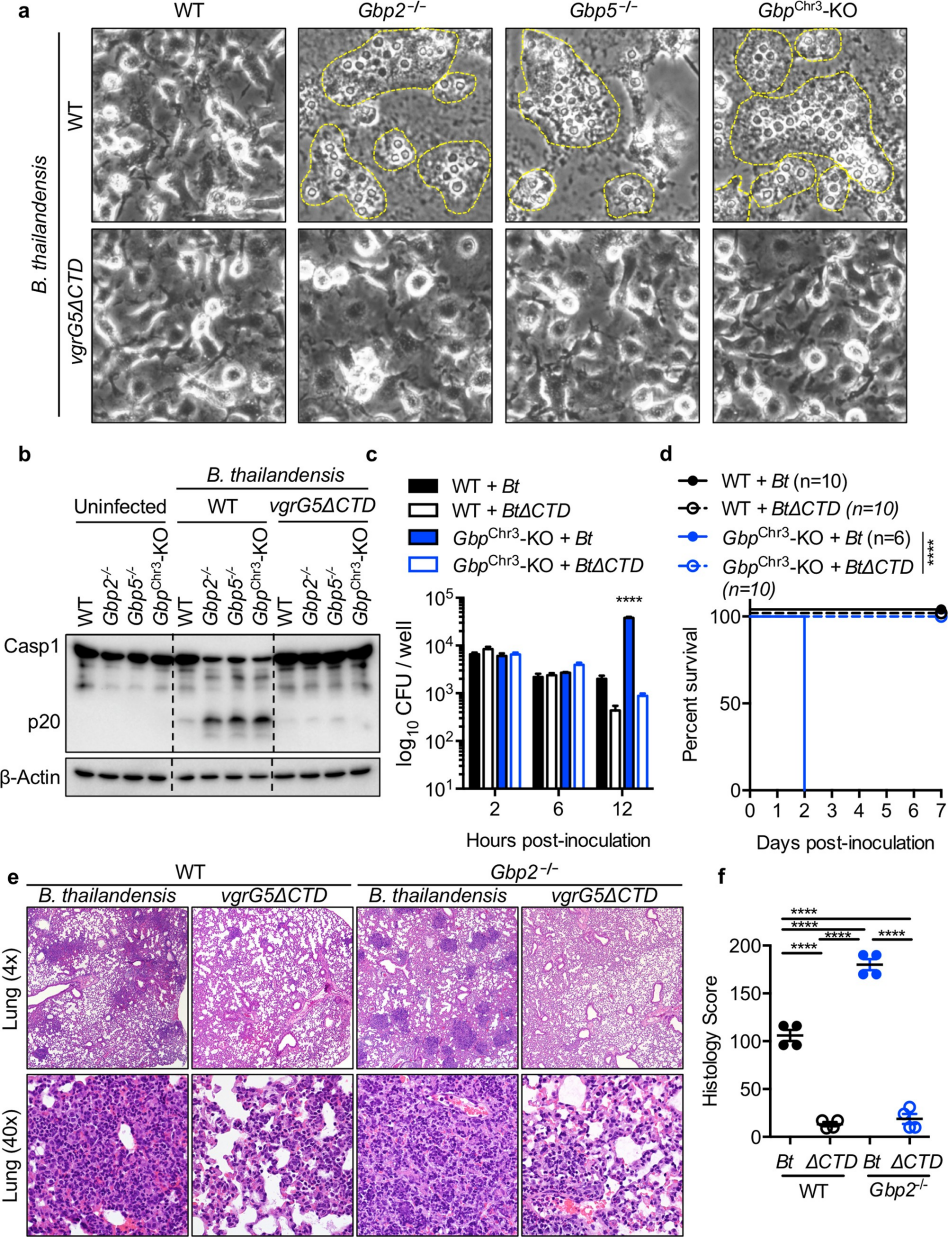
VgrG5-dependent fusion required for increased pathogenesis in GBP deficiency. Unprimed BMDMs were infected (MOI 5) with *B*. *thailandensis* (WT or *vgrG5ΔCTD*) and (**a**) phase contrast images (20x) were taken after 20 h to observe the extent of MNGC fusion. (**b**) Immunoblots were probed for caspase-1 cleavage following infection with *B*. *thailandensis* (WT or *vgrG5ΔCTD*). (**c**) BMDMs were infected with *B*. *thailandensis* (WT or *vgrG5ΔCTD*) and intracellular bacterial CFUs were determined by serial dilution of lysed cells at the indicated time points. (**d**) Indicated mouse strains were inoculated (i.n.) with *B*. *thailandensis* (WT or *vgrG5ΔCTD*) (1 x 10^5^) and monitored for survival following infection for 7 days. (**e,f**) Wildtype and *Gbp2*^**−/−**^ mice were inoculated with *B*. *thailandensis* (WT or *vgrG5ΔCTD*, 5 x 10^3^) and lungs were fixed in formalin, stained with H&E, and scored (described in Methods) by a trained pathologist (PV). Statistical significance was determined by Dunnet’s multiple comparison test (**c**), the log-rank test (**d**), or one-way ANOVA with Tukey’s multiple comparison test (**f**), ****P < 0.0001. Data are representative of (**a,b,c**) at least three independent experiments, (**d**) pooled from two independent repeats, or (**e,f**) a single experiment.

Given our findings that GBPs inhibit actin polymerization-dependent MNGC fusion in vitro, we hypothesized that loss of GBPs and increased cell-cell fusion would render mice highly susceptible to *B*. *thailandensis*. Consistent with our hypothesis, intranasal infection of mice with *B*. *thailandensis* revealed that *Gbp2*^**−/−**^, *Gbp5*^**−/−**^, and *Gbp*^Chr3^-KO mice were highly susceptible to infection (**Fig 5A–5C**). Furthermore, GBP-deficient mice harbored increased numbers of bacteria in the lungs and spleen (**Fig 5D and 5E**) and exhibited an increase in inflammatory pathology characterized by conspicuous multifocal lesions, increased interstitial inflammation, and edema (**Fig 5F and 5G**). These findings are consistent with our observation that inflammasome activation is increased during *B*. *thailandensis* infection of BMDMs, likely as a consequence of increased cell-cell spread and bacterial replication (**Fig 2F**) and previous studies showing that lethal pathology is driven by excessive IL-1β production during *B*. *pseudomallei* respiratory infection [8]. Because *B*. *thailandensis* induces inflammasome activation in BMDMs independent of GBPs (**Fig 2F**), we hypothesized that the larger infected cell mass following MNGC formation would lead to increased activation of the inflammasome and allow for greater replication of *B*. *thailandensis*. To further confirm whether bacterial VgrG5 was required to drive the increased fusion observed in *Gbp2*^**−/−**^ and *Gbp5*^**−/−**^ macrophages (**Fig 2D**), we infected BMDMs with wildtype *B*. *thailandensis* or *B*. *thailandensis vgrG5ΔCTD*, a mutant of VgrG5 that is unable to induce cell-cell fusion [6,7]. Consistent with our previous results, we found that the VgrG5 CTD was required for the increased MNGC formation and inflammasome activation observed in GBP-deficient BMDMs (**Fig 6A and 6B**). To determine whether GBPs restrict bacterial replication through restriction of cell fusion, we infected wildtype or *Gbp*^Chr3^-KO BMDMs with wildtype or the fusion-defective *B*. *thailandensis vgrG5ΔCTD* and quantified intracellular bacteria after invasion (2 h), at the start of cell fusion (6 h), or after the initiation of fusion (12 h). We find similar numbers of intracellular bacteria after entry (2 h) and at the start of cell fusion (6 h) and VgrG5 CTD-dependent increases in bacterial replication in GBP-deficient macrophages (12 h) (**Fig 6C**). Infection of wildtype and *Gbp*^Chr3^-KO mice with wildtype or fusion-defective *B*. *thailandensis vgrG5ΔCTD* further substantiated our hypothesis that increased cell-cell fusion, bacterial replication, and VgrG5-induced pathology contribute to the susceptibility of GBP-deficient mice (**Fig 6D).** Histological analysis of infection in wildtype and *Gbp2*^**−/−**^ mice with wildtype *B*. *thailandensis* or *B*. *thailandensis vgrG5ΔCTD* (5 x 10^3^) showed that the increased pathology and bacterial replication observed in *Gbp2*^**−/−**^ mice requires the VgrG5 CTD (**Fig 6E and 6F, S6A and S6B Fig**). Infection with a 200-fold higher dose (1 x 10^6^) was largely similar in terms of survival, although *Gbp*^Chr3^-KO mice were slightly more susceptible to infection by *B*. *thailandensis vgrG5ΔCTD* than *Gbp2*^**−/−**^ and *Gbp5*^**−/−**^ mice, suggesting that at this high dose GBPs may play a small role outside of their function to restrict cell-cell fusion-induced pathology (**S6 Fig**). Overall, our results suggest that GBPs are required to restrict *B*. *thailandensis* cell fusion-mediated pathogenesis in vivo.

## Discussion

In this study, we showed that GBPs are essential for controlling *B*. *thailandensis* infection and that they act by restricting bacterial actin-based motility and fusion with neighboring cells, a unique strategy utilized by *Burkholderia spp*. (**S7 Fig**) [11,30]. Both the GTPase activity and CAAX prenylation domain of GBP2 were required for recruitment to *B*. *thailandensis*. Actin polymerization was restricted on bacteria coated with GBP2, suggesting GBPs directly or indirectly inhibit bacteria-mediated actin polymerization in infected cells. Mice lacking GBP2, GBP5, or multiple GBPs on chromosome 3 (GBP2, GBP2b (GBP1), GBP3, GBP5, GBP7) were significantly more susceptible to infection, revealing a critical role for these proteins in restricting bacterial infection. This susceptibility is in contrast to previous work showing that wildtype and *Gbp*^Chr3^-KO mice were similar in their control of bacterial burden following intraperitoneal *B*. *thailandensis* infection, possibly due to the use of an intraperitoneal infection model [31]. Interestingly, the unique virulence strategy of fusion with neighboring cells used by *B*. *thailandensis*, which is shared with other members of the *B*. *pseudomallei* group and mediated by the bacterial protein VgrG5, was required to drive pathogenesis in cells and mice lacking these GBP proteins.

Earlier studies with human GBP1 revealed that GBP1 can bind globular actin and inhibit the polymerization of filamentous F-actin fibers [14]. More recently, human GBP1 was shown to inhibit actin dynamics during KSHV and *Shigella* infection [15,17,26]. The close association of GBP2 with non-motile intracellular *B*. *thailandensis* would suggest GBP2 directly, or through recruitment of accessory actin regulators, can limit bacteria-mediated actin polymerization, but the mechanism is still poorly understood. Human GBP1 was also shown to recruit to *B*. *thailandensis*, but no role for this association was described [15]. Comparatively less is known about how the murine GBPs function in regulating actin dynamics. The human and murine GBPs containing a CAAX domain (GBP1 (GBP2b), GBP2, GBP5) differ in their activity due to unique evolution with their associated pathogens [32,33]. Indeed, human GBP1 requires a triple-arginine motif for recruitment to *S*. *flexneri* and subsequent recruitment of GBP2, GBP3, GBP4, and GBP6, while murine GBPs lack this motif [15]. Pathogen virulence factors also determine the activity of GBPs, with *S*. *flexneri* secreting IpaH9.8, a ubiquitin ligase that targets a subset of GBPs for proteasomal degradation while leaving others [15,17,27]. Host specificity of pathogens also regulates the recruitment of GBPs, as *C*. *muridarum* fails to become targeted by murine GBP2 while human-adapted *C*. *trachomatis* does [33]. These differences between host and pathogen highlight the importance of using multiple model organisms to study cellular immunity.

During some intracellular infections, GBPs are recruited to pathogen-containing vacuoles, as observed during *Toxoplasma*, *Mycobacterium bovis*, and *C*. *trachomatis* infection, or directly to bacterial membranes in the case of cytosolic *Francisella* or *Shigella* infection [3,20,21,34,35]. Models suggest GBPs can restrict pathogens by exposing bacterial ligands from pathogen-containing vacuoles or directly from cytosolic bacteria for sensing by the inflammasome [19–21,36]. Murine GBP2 has been shown to promote caspase-11 and caspase-1 inflammasome activation in response to Gram-negative bacteria and intracellular LPS [36,37]. Far less is known about the role of murine GBP5 in bacterial immunity except for its role in activation of caspase-1 during *F*. *novicida* infection [20]. Early studies suggested that GBP5 is important for *S*. Typhimurium inflammasome activation and activation of the NLRP3 inflammasome, but follow-up studies using independently generated knockout mice on the C57BL6 background indicate that these original findings should be re-examined [2,36,38–40]. Newer work suggests GBP5 may be important for activation of the inflammasome by bacterial OMVs [41]. Interestingly, GBPs are not required for *B*. *thailandensis* inflammasome activation in unprimed BMDMs, suggesting GBPs are not efficient at killing *B*. *thailandensis*, similar to previous findings with *S*. *flexneri* [15]. Of note, previous work found GBPs were not required for inflammasome activation during *B*. *thailandensis* infection in BMDMs using a different method of infection than used in this study [36]. Additionally, *B*. *thailandensis* does not target GBPs for proteasome degradation, as observed during *S*. *flexneri* infection [17,27]. These findings suggest that *B*. *thailandensis* is generally well controlled by cell-autonomous immunity in mammals. While GBPs appear to play a minor role in direct promotion of inflammasome activation, previous studies have established an important role for many inflammasome signaling components and the downstream inflammatory cytokines in both protection and immunopathology during *B*. *thailandensis* and *B*. *pseudomallei* infections [8–10,31,42]. Many pathogens encode bacterial factors that nucleate actin polymerization that is dependent upon the Arp2/3 complex [43]. While *B*. *thailandensis* also utilizes the host Arp2/3 actin polymerization machinery, the more virulent species *B*. *pseudomallei* and *B*. *mallei* mimic Ena/VASP (vasodilator-stimulated phosphoprotein) actin nucleation factors for motility in eukaryotic cells, suggesting their relatively increased virulence may be a result of escaping GBP-mediated Arp2/3-dependent actin inhibition [11,22–24]. Unlike in *Listeria* infection, where overexpression of the Arp2/3-binding domain of Scar1 inhibits motility, *B*. *pseudomallei* actin tail formation was not inhibited, suggesting there are unique pathogen-specific mechanisms for hijacking host actin polymerization and different host factors that are required to restrict motility [24]. Functionally, BimA can be swapped between the three *B*. *pseudomallei* group species and restore motility; however, BimA from *B*. *pseudomallei* and *B*. *mallei* generate distinct F-actin tails from that of *B*. *thailandensis* due to differences in their BimA protein-protein interaction domains [11,22,23]. Given the diversity between the *B*. *pseudomallei* group members, other actin hijacking pathogens likely have an even greater diversity in how they manipulate host actin and how the host immune system responds. Further studies on GBPs will help to reveal more about their mechanism of action during infection and how they mechanistically inhibit actin polymerization. As an intracellular bacterium that navigates from the endosomal compartment to the cytosol before driving fusion with neighboring cells, *B*. *thailandensis* is a useful model organism for studying how cell-autonomous immunity controls a dynamic pathogen that hijacks multiple eukaryotic cellular systems.

## Materials and methods

### Mice

Wildtype (C57BL6/J), *Gbp2*^−/−^ (*Gbp2*^tm1b(KOMP)Wtsi^*)*, *Gbp5*^−/−^, *Gbp*^Chr3^-KO (MGI:5438974*)*, *Irgb10*^*-/-*^, *Trif*^−/−^ (*Ticam1*^tm1Aki^*)*, *Myd88*^−/−^(*Myd88*^tm1Aki^*)*, *Trif*^−/−^*Myd88*^−/−^, *Irf3*^−/−^(*Irf3*^tm1Ttg^), *Irf7*^−/−^ (*Irf7*^tm1Ttg^*)*, *Irf3*^−/−^*Irf7*^−/−^, *Ifnar1*^−/−^(*Ifnar1*^tm1Agt^*)*, *Ifnar2*^−/−^ (*Ifnar2*^tm1Pjh^*)*, *Irf9*^−/−^ (*Irf9*^tm1Ttg^*)*, *Irf1*^−/−^ (*Irf1*^tm1Mak^*)*, *Mb21d1*^−/−^ (cGAS, 026554, The Jackson Laboratory), *Sting*^−/−^ (*Tmem173*^*gt*^, 017537, The Jackson Laboratory), *Mavs*^−/−^(*Mavs*^tm1Aki^*)*, *Ripk2*^−/−^ (*Ripk2*^tm1Flv^*)*, *Tlr2*^−/−^ (*Tlr2*^tm1Aki^*)*, *Tlr3*^−/−^ (*Tlr3*^tm1Flv^), *Tlr4*^−/−^ (*Tlr4*^tm1Aki^*)*, and *Tlr5*^−/−^ (008377, The Jackson Laboratory) mice have been described previously [19,20,36]. Male and female mice between 6–8 weeks old were used in this study. Mice were bred at St. Jude Children’s Research Hospital and studies were conducted under protocols approved by St. Jude Children’s Research Hospital Institutional Committee on the Use and Care of Animals.

### Bone Marrow-Derived Macrophage Culture

Primary bone marrow–derived macrophages (BMDMs) were grown for 6 days in DMEM (11995073, Thermo Fisher Scientific, Waltham, MA) supplemented with 10% FBS (TMS-013-B, Millipore, Billerica, MA or S1620, BioWest, Riverside, MO), 30% L929-conditioned media and 1x penicillin-streptomycin (15070063, Thermo Fisher Scientific). BMDMs were seeded in antibiotic–free media at a concentration of 1 x 10^6^ cells onto 12-well plates and incubated overnight. Cells were further washed and cultured in antibiotic-free DMEM with 10% FBS before stimulations or infections.

### Lentiviral GBP2 transduction

The N-terminal mCherry-GBP2 fusion constructs were constructed using the NEBuilder HiFi DNA assembly kit (E2621S, New England BioLabs) according to the manufacturer’s protocol. PCR products were generated for mCherry, wildtype GBP2, or GBP2^ΔCAAX^ and assembled with the vector backbone pCDH-CMV-MCS-EF1a-GFP-Neo (modified from CD516B-2, System Biosciences) digested by EcoRI and treated with phosphatase to prevent re-ligation. Site-directed mutagenesis was utilized to generate the mCherry-GBP2^K51A^ plasmid (E0554S, New England BioLabs). To generate lentiviral particles, plasmids psPAX2 (12260, Addgene), pMD2.G (12259, Addgene), and the mCherry-GBP–containing transfer plasmids were co-transfected into 293T cells. Viral particle-containing supernatant plus polybrene (6 μg/mL) was used to transduce NIH3T3 fibroblasts. Transduced cells were selected with G418 (400 μg/mL, A1720, Sigma-Aldrich) to generate fibroblasts stably expressing mCherry-tagged GBP2 proteins.

### Bacterial Culture

*B*. *thailandensis* strain E264 (GFP^+^) and *B*. *thailandensis vgrG5ΔCTD* (vgrG5 lacking the C-terminal domain required for fusion activity [6]), provided by Dr. Joseph Mougous (University of Washington, Seattle, WA), were cultured in LB broth (3002–031, MP Biomedicals, Santa Ana, CA) overnight and subcultured into fresh LB media for 3 h at 37°C to generate log-phase grown bacteria.

### Mouse infections

*B*. *thailandensis* was grown as described above. For mouse infection experiments, quantified frozen aliquots of *B*. *thailandensis* were diluted in PBS before infection to inoculate at 5 x 10^3^ bacteria per mouse unless otherwise indicated. Mice were anesthetized with isoflurane and administered *B*. *thailandensis* in a 50 μL PBS suspension via the nares. After two days, lungs and spleens were harvested for histology (tissues fixed in formalin and processed by the St. Jude Children’s Research Hospital Veterinary Pathology Core) or colony forming unit (CFU) analysis. Histological scoring was performed by a board certified veterinary pathologist (PV) and assigned a semi-quantitative score based on the following severity grades (grade = semi-quantitative score): (0 = 0) within normal limits, (1 = 1/1.5 = 8) minimal: rare or inconspicuous lesions, (2 = 15/2.5 = 25) mild: multifocal or small, focal, or widely separated but conspicuous lesions, (3 = 40/3.5 = 60) moderate: multifocal, prominent lesions, (4 = 80/4.5 = 90) marked: extensive to coalescing lesions or areas of inflammation with some loss of structure, and (5 = 100) severe: diffuse lesion with effacement of normal structure. CFUs were determined by homogenizing tissue in PBS with metal beads for 2 min using the QIAGEN TissueLyser II apparatus and plating on LB agar plates incubated overnight at 37°C.

### Bone marrow-derived macrophage stimulations

BMDMs were differentiated as described above. Prior to stimulation, cells were rinsed with PBS, and PBS was replaced with fresh antibiotic-free DMEM containing 10% FBS. *B*. *thailandensis* (MOI 5) was pelleted onto cells at 300 x g for 5 min; cells were washed after 1 h, incubated with DMEM (1000 μg/mL kanamycin) for 1 h, washed, and finally incubated in DMEM (250 μg/mL kanamycin) for the remainder of the experiment. After the final wash, the following were added as indicated: SYTOX Green (25 nM, S7020, Thermo Fisher Scientific), CK-666 (200 μM, SML0006, Sigma), IFNγ (100 ng/mL, 315–05, Peprotech), IFNβ (100U/mL, 12401–1, PBL Assay Science). Unless otherwise indicated phase-contrast images were collected at 20 h post-infection. To determine intracellular CFUs, cells were washed with PBS, lysed with PBS containing 0.01% Triton X-100, serially diluted in PBS, and plated on LB agar plates incubated at 37°C.

### Microscopy methods

For live cell imaging experiments to detect cell-cell fusion events, cells were stained with CellTrace Violet (C34557, Thermo Fisher Scientific) or CellTrace Far Red (C34564, Thermo Fisher Scientific) following manufacturer’s protocol and mixed at a 1:1 ratio prior to seeding 0.8 x 10^6^ BMDMs or 1 x 10^5^ NIH3T3 cells per chamber of a 4-chamber μ-slide (80426, Ibidi). Chambers were infected as described above, and following the final wash, media containing kanamycin (250 μg/mL) and Sytox Green (25 nM) was added and the chamber slide cover was replaced by a sterile differential interference contrast (DIC)-compatible lid (80055, Ibidi) for live imaging with fluorescence and DIC. Colocalization analysis was performed using the ImarisColoc software (Oxford Instruments, Zurich, Switzerland). For fluorescence microscopy experiments, BMDMs and NIH3T3 cells were fixed in 4% PFA, washed with PBS, permeabilized with 0.1% Triton X-100, and stained with phalloidin-iFluor555 (ab176756, Abcam), phalloidin-iFluor647 (ab176759, Abcam), and DAPI, and coverslips were mounted in ProLong Diamond with DAPI (P36962, Thermo Fisher Scientific). For confocal images, a Nikon C2 microscope was used; for super-resolution structured illumination microscopy, a Zeiss Elyra PS.1 microscope was used as previously described [19]. Quantitative cell death measurements by Sytox Green uptake counts were collected hourly on an IncuCyte S3 system (Essen BioScience). For transmission electron microscopy images, BMDMs were fixed, processed, and imaged on an FEI Helios NanoLab focused ion beam scanning electron microscope (FIB-SEM), with images from a single plane stitched together using the MAPS software package (FEI).

### Immunoblotting analysis

For signaling blots, supernatant was removed, and cells were lysed in RIPA buffer containing protease and phosphatase inhibitors plus 4x Laemmli sample buffer. Caspase-1 cleavage was measured from the combined cell lysate and supernatants. Proteins were separated via SDS-PAGE with 6–12% polyacrylamide gels, transferred to PVDF membranes (IPVH00010, Millipore), and blocked with 5% nonfat dry milk. Primary antibodies against caspase-1 (AG-20B-0042-C100, Adipogen), GBP2 (11854-1-AP, Proteintech), GBP5 (13220-1-AP, Proteintech), or β-actin (8457, Cell Signaling Technologies) were incubated overnight at 4°C followed by appropriate secondary antibodies conjugated with HRP incubated for 1 h at room temperature (Jackson ImmunoResearch, West Grove, PA). Membranes were visualized using Luminata Forte Chemiluminescence substrate (WBLUF0500, Millipore) on a BioRad ChemiDoc.

### Microarray analysis

BMDMs were infected as described above, and RNA was collected at 3 h post-infection with Trizol (Ambion). Total RNA was submitted to the Hartwell Center core facility, and 200 ng total RNA was processed using the Affymetrix WT Plus protocol for use with the Mouse Clariom S Gene Chip array (902930, Thermo Fisher Scientific). Differential expression was defined by a 0.5-fold (log_2_ signal) change between conditions.

### Quantification and statistical analysis

GraphPad Prism 6.0 software was used for data analysis. Data are shown as mean ± SEM. Statistical significance was determined by the Mann-Whitney test for two groups or one-way ANOVA for three or more groups with the Dunn’s or Holm-Sidak multiple comparison test. The specific statistical testing for each experiment is indicated in the figure legends. Survival curves were compared using the log-rank test. *P* < 0.05 was considered statistically significant.

### Ethics statement

Studies were conducted under protocols approved by St. Jude Children’s Research Hospital Institutional Committee on the Use and Care of Animals, protocol number 482. It is the policy of the St. Jude Children's Research Hospital Animal Care and Use Committee that all research involving animals be conducted according to the highest possible professional, ethical, and scientific standards and that all animals be housed, maintained, and handled in compliance with the standards set forth by the Animal Welfare Act of 1966 (9 CFR Part 3 as amended); the National Research Council 1996 “Guide for the Care and Use of Laboratory Animals”; the Public Health Service Policy on the Humane Care and Use of Laboratory Animals (revised September 1986); the United States Government Principles for the Utilization and Care of Vertebrate Animals Used in Testing, Research, and Training; the report of the American College of Laboratory Animal Medicine on Adequate Veterinary Care in Research, Testing, and Teaching; the 2007 Guidelines on Euthanasia; and all other applicable federal, state, and local laws, regulations, and policies. St. Jude Children's Research Hospital is committed to maintaining full accreditation status by AAALAC International.

## Supporting information

S1 Fig IFN-inducing intracellular receptors are not required for restricting *B*. *thailandensis* MNGC formation. Unprimed BMDMs were infected with *B*. *thailandensis* (MOI 5), and images were collected at 20 h post-infection. Images are representative of two independent experiments. Refers to Fig 1.
(TIFF)

S2 Fig IRGB10 is not required for restriction of *B*. *thailandensis*. BMDMs were infected with *B*. *thailandensis* and (**a**) phase-contrast images were collected at 20 h post-infection and (**b**) caspase-1 immunoblots performed on lysates collected 20 h post-infection. Images are representative of at least two independent experiments. Refers to Fig 2.
(TIFF)

S3 Fig Ultrastructure of wildtype and *Gbp*^Chr3^-KO BMDMs infected with *B*. *thailandensis*. Unprimed BMDMs were infected with *B*. *thailandensis* for 3 h, and **(a)** wildtype BMDMs and **(b)** *Gbp*^Chr3^-KO BMDMs were fixed and processed to examine changes in ultrastructure during fusion. Black bars measure 7 μm (low magnification) or 2 μm (high magnification). Black arrows indicate *B*. *thailandensis*. Images are representative of two independent experiments. Refers to Fig 2.
(TIFF)

S4 Fig IFN priming does not protect *Gbp*^Chr3^-KO BMDMs from fusion and increased pyroptosis. **(a,b)** Unprimed BMDMs were infected with *B*. *thailandensis* (MOI 5), and Sytox Green uptake was measured over time. **(c)** Primed (16 h) BMDMs were infected, and images were collected at 20 h post-infection. **(d)** Caspase-1 (CASP1) cleavage was measured at 20 h. **(e)** Cell death in primed cells was monitored by Sytox Green uptake. Data are representative of three independent experiments. Statistical significance was determined by Dunnet’s multiple comparison test **(a,b,e)**, ****P < 0.00001. Refers to Fig 2.
(TIFF)

S5 Fig Amino acid alignment of CAAX box protein C-terminal domains. Amino acid sequences from the C-terminus of Rho, Ras, and GBP family proteins were aligned by CLUSTAL Omega (EMBL-EMI) and visualized in AliView with the ClustalX color scheme (http://ormbunkar.se/aliview/). The triple-arginine motif in human Gbp1 is outlined in red to highlight that the other GBPs lack this motif. The carboxyl-terminal CAAX box is highlighted to show conservation between GBPs and the small GTPases, which regulate actin dynamics. This conserved domain is post-translationally modified by prenylation on the conserved cysteine and cleavage of the final three amino acids, allowing these proteins to associate with membranes. Refers to Figs 2 and 4.
(TIFF)

S6 Fig *B*. *thailandensis* VgrG5-mediated fusion drives bacterial replication and mortality in GBP-deficient mice. Mice were inoculated intranasally with *B*. *thailandensis* (WT or *vgrG5ΔCTD*). **(a,b)** *B*. *thailandensis* (5 x 10^3^)-infected mice at day 2 post-infection were used to quantify bacterial colony-forming units (CFUs) in the lungs and spleen by serially diluting and plating. (**c**) Survival following a high dose infectious challenge with *B*. *thailandensis* (1 x 10^6^) was monitored in the indicated knockout mice. Statistical significance was determined by (**a,b**) one-way ANOVA with Tukey’s multiple comparison test or (**c**) the log-rank test, n.s. not significant, *P < 0.05,**P < 0.001, ****P < 0.00001. Data are representative of a single experiment (**a,b**) or pooled from two experiments (**c**). Refers to Fig 6.
(TIFF)

S7 Fig Working model for GBP-mediated inhibition of *B*. *thailandensis* actin-mediated cell-cell fusion. (TIFF)

S1 Video Cell fusion is restricted in wildtype BMDMs during *B*. *thailandensis* infection. Video was constructed from confocal images collected every 45 min on a Nikon C2 microscope in Nikon Elements software. Unprimed wildtype BMDMs were stained with CellTrace Far Red or CellTrace Violet and mixed at a 1:1 ratio before seeding on Ibidi coverslips. Sytox Green (25 nM) was added after final washes to stain nuclei of permeabilized cells. Video is representative of three independent fields of view. Video refers to data in Fig 2.
(MOV)

S2 Video Cell fusion is increased in *Gbp*^Chr3^-KO BMDMs during *B*. *thailandensis* infection. Video was constructed from confocal images collected every 45 min on a Nikon C2 microscope in Nikon Elements software. Unprimed *Gbp*^Chr3^-KO BMDMs were stained with CellTrace Far Red or CellTrace Violet and mixed at a 1:1 ratio before seeding on Ibidi coverslips. Sytox Green (25 nM) was added after final washes to stain nuclei of permeabilized cells. Video is representative of three independent fields of view. Video refers to data in Fig 2.
(MOV)
