# Supplementary figures and images for: Interferon inducible GBPs restrict Burkholderia thailandensis motility induced cell-cell fusion
Source: PLoS Pathog. 2020 Mar 9;16(3):e1008364. doi: 10.1371/journal.ppat.1008364 (PMC7082077; doi:10.1371/journal.ppat.1008364)

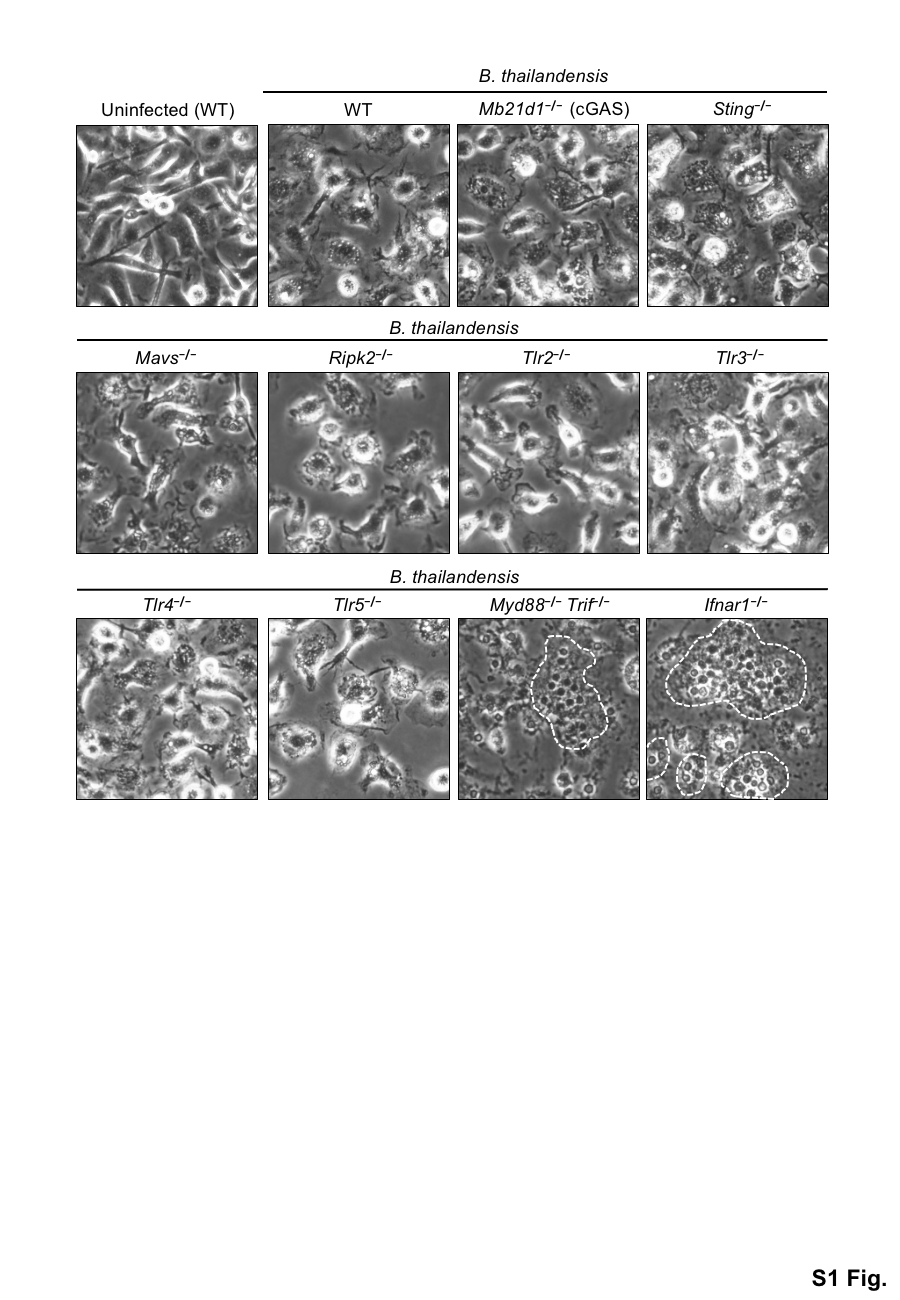

Supplement: S1 Fig — Unprimed BMDMs were infected with B. thailandensis (MOI 5), and images were collected at 20 h post-infection. Images are representative of two independent experiments. Refers to Fig 1. (TIFF) [file ppat.1008364.s001.tiff]

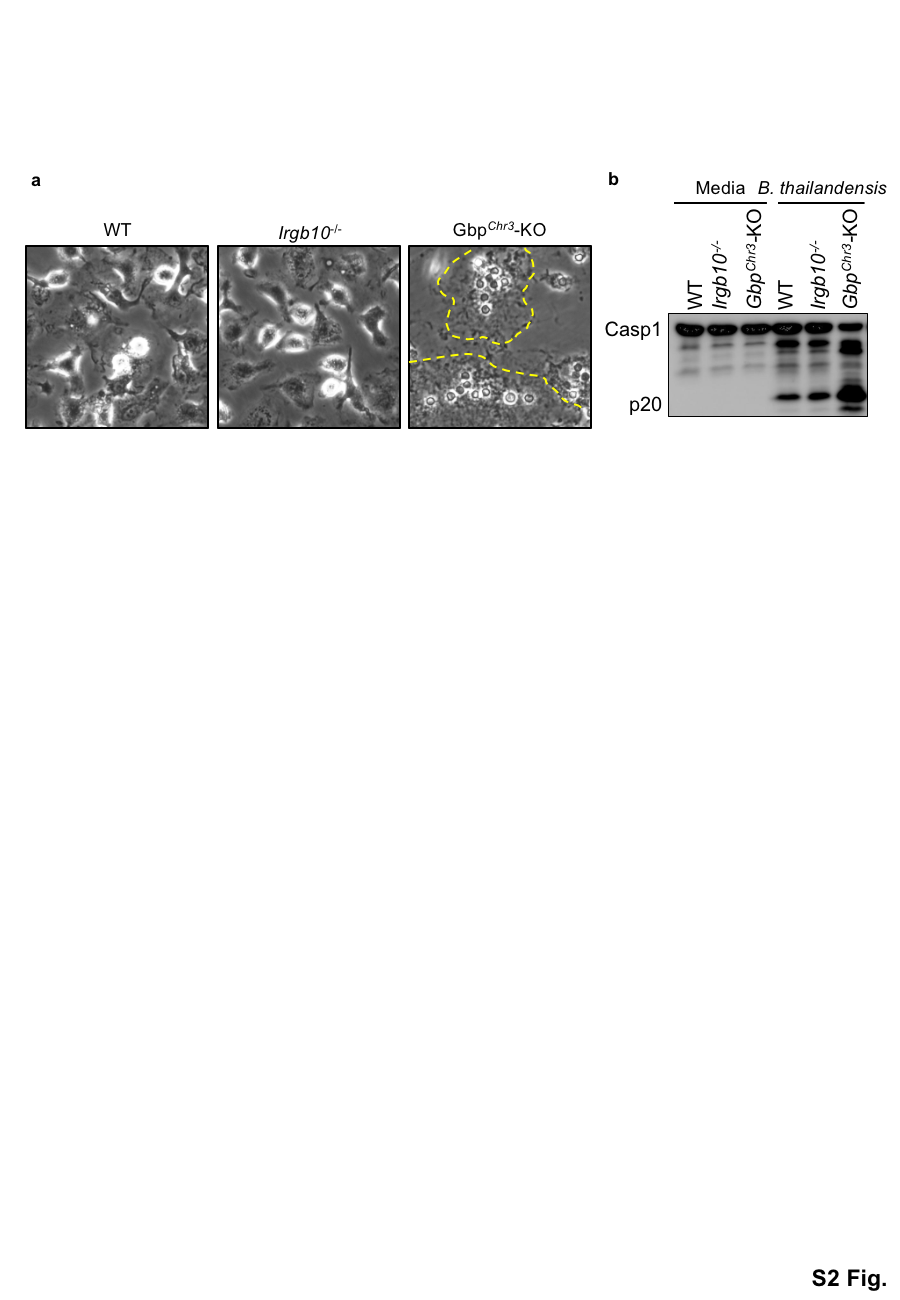

Supplement: S2 Fig — BMDMs were infected with B. thailandensis and (a) phase-contrast images were collected at 20 h post-infection and (b) caspase-1 immunoblots performed on lysates collected 20 h post-infection. Images are representative of at least two independent experiments. Refers to Fig 2. (TIFF) [file ppat.1008364.s002.tiff]

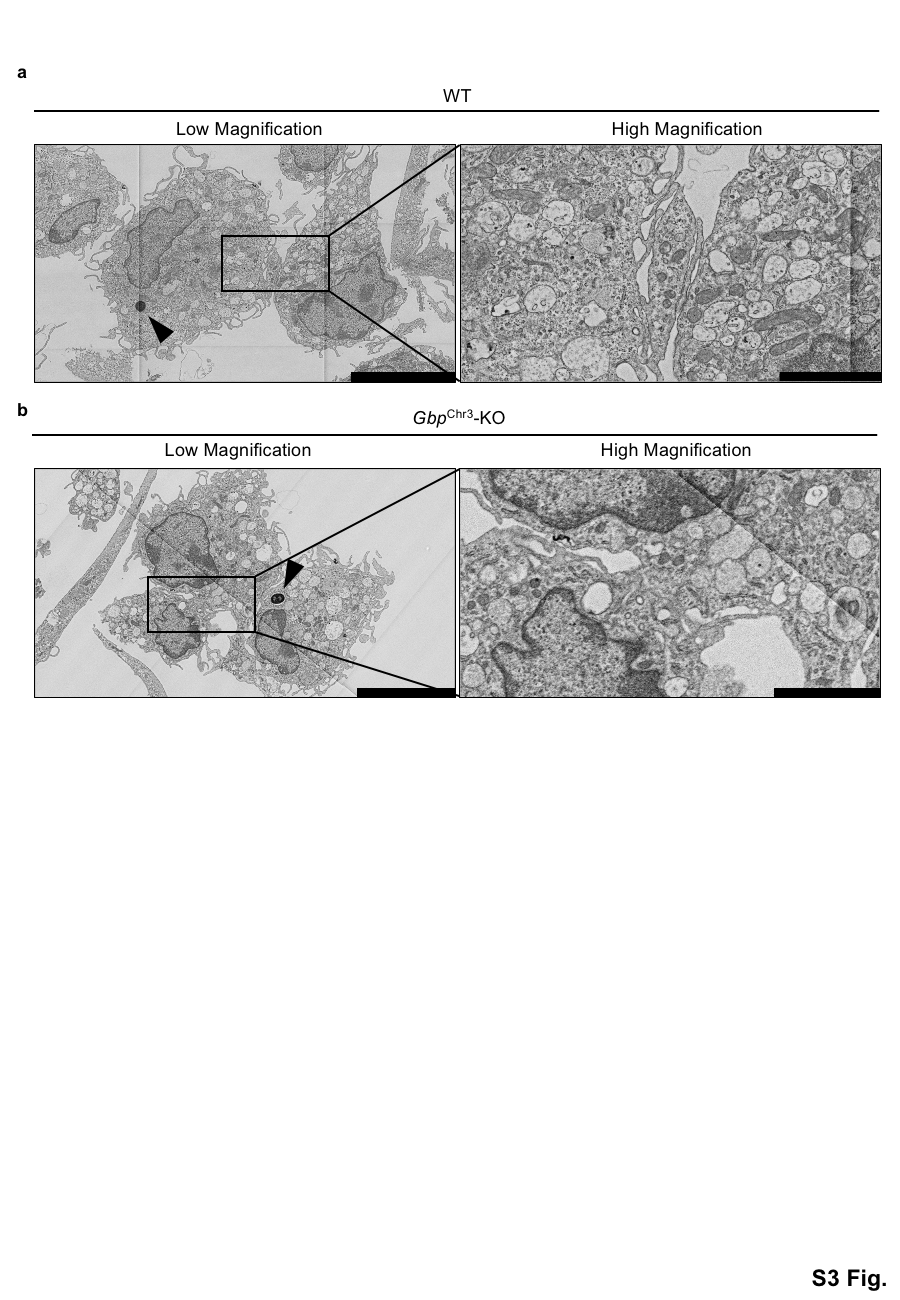

Supplement: S3 Fig — Unprimed BMDMs were infected with B. thailandensis for 3 h, and (a) wildtype BMDMs and (b) GbpChr3-KO BMDMs were fixed and processed to examine changes in ultrastructure during fusion. Black bars measure 7 μm (low magnification) or 2 μm (high magnification). Black arrows indicate B. thailandensis. Images are representative of two independent experiments. Refers to Fig 2. (TIFF) [file ppat.1008364.s003.tiff]

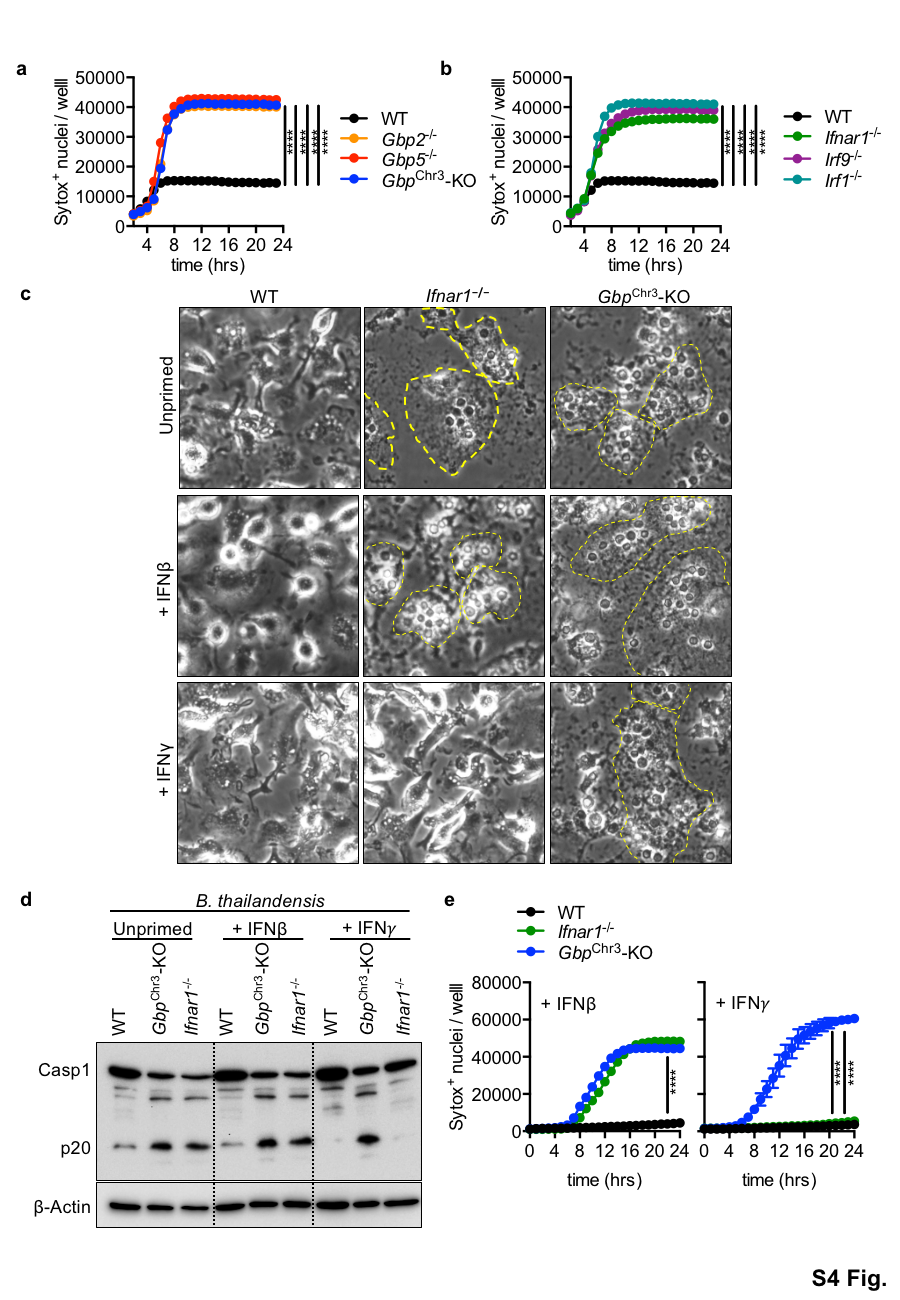

Supplement: S4 Fig — (a,b) Unprimed BMDMs were infected with B. thailandensis (MOI 5), and Sytox Green uptake was measured over time. (c) Primed (16 h) BMDMs were infected, and images were collected at 20 h post-infection. (d) Caspase-1 (CASP1) cleavage was measured at 20 h. (e) Cell death in primed cells was monitored by Sytox Green uptake. Data are representative of three independent experiments. Statistical significance was determined by Dunnet’s multiple comparison test (a,b,e), ****P < 0.00001. Refers to Fig 2. (TIFF) [file ppat.1008364.s004.tiff]

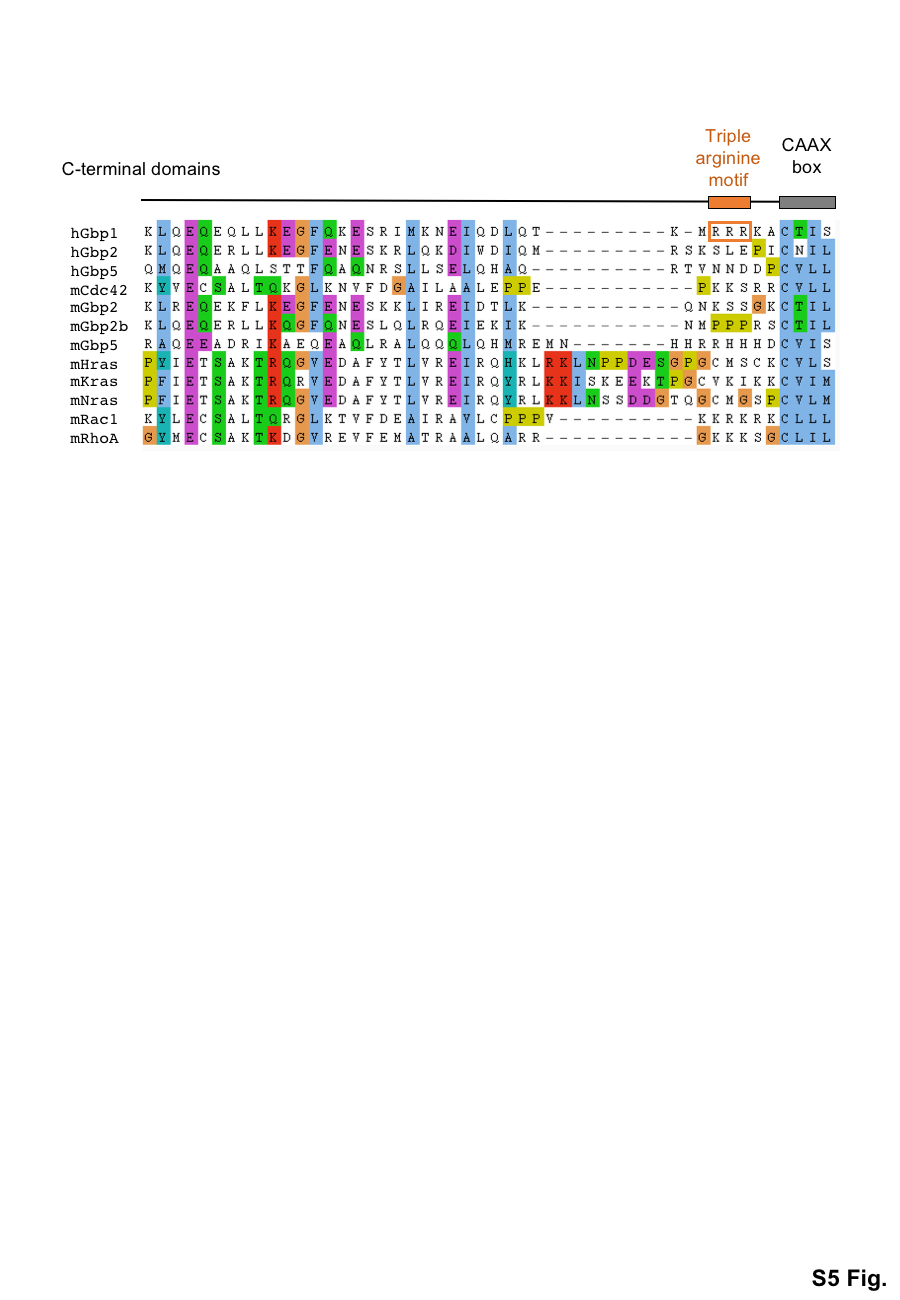

Supplement: S5 Fig — Amino acid sequences from the C-terminus of Rho, Ras, and GBP family proteins were aligned by CLUSTAL Omega (EMBL-EMI) and visualized in AliView with the ClustalX color scheme (http://ormbunkar.se/aliview/). The triple-arginine motif in human Gbp1 is outlined in red to highlight that the other GBPs lack this motif. The carboxyl-terminal CAAX box is highlighted to show conservation between GBPs and the small GTPases, which regulate actin dynamics. This conserved domain is post-translationally modified by prenylation on the conserved cysteine and cleavage of the final three amino acids, allowing these proteins to associate with membranes. Refers to Figs 2 and 4. (TIFF) [file ppat.1008364.s005.tiff]

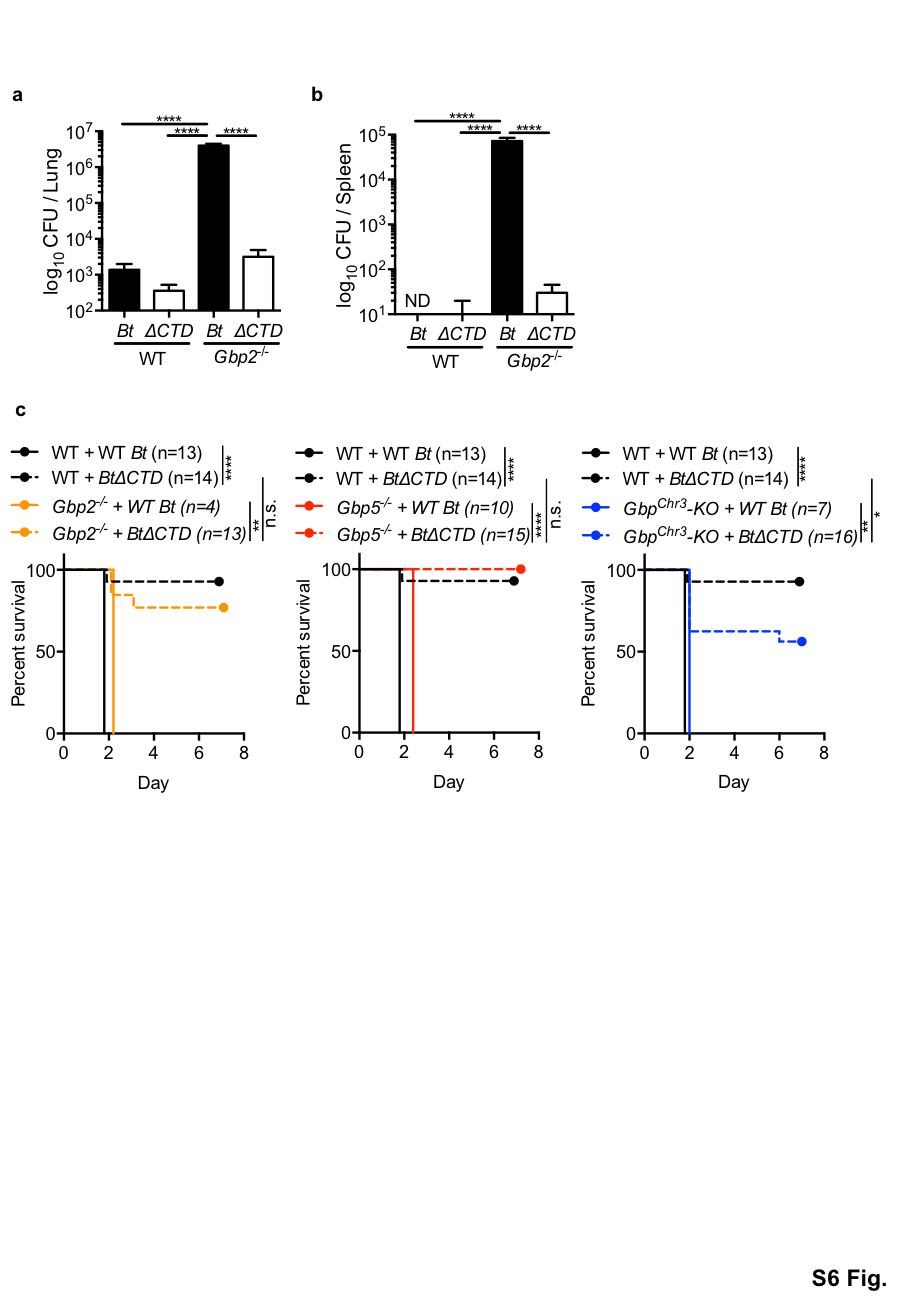

Supplement: S6 Fig — Mice were inoculated intranasally with B. thailandensis (WT or vgrG5ΔCTD). (a,b) B. thailandensis (5 x 10^3^)-infected mice at day 2 post-infection were used to quantify bacterial colony-forming units (CFUs) in the lungs and spleen by serially diluting and plating. (c) Survival following a high dose infectious challenge with B. thailandensis (1 x 10^6^) was monitored in the indicated knockout mice. Statistical significance was determined by (a,b) one-way ANOVA with Tukey’s multiple comparison test or (c) the log-rank test, n.s. not significant, *P < 0.05,**P < 0.001, ****P < 0.00001. Data are representative of a single experiment (a,b) or pooled from two experiments (c). Refers to Fig 6. (TIFF) [file ppat.1008364.s006.tiff]

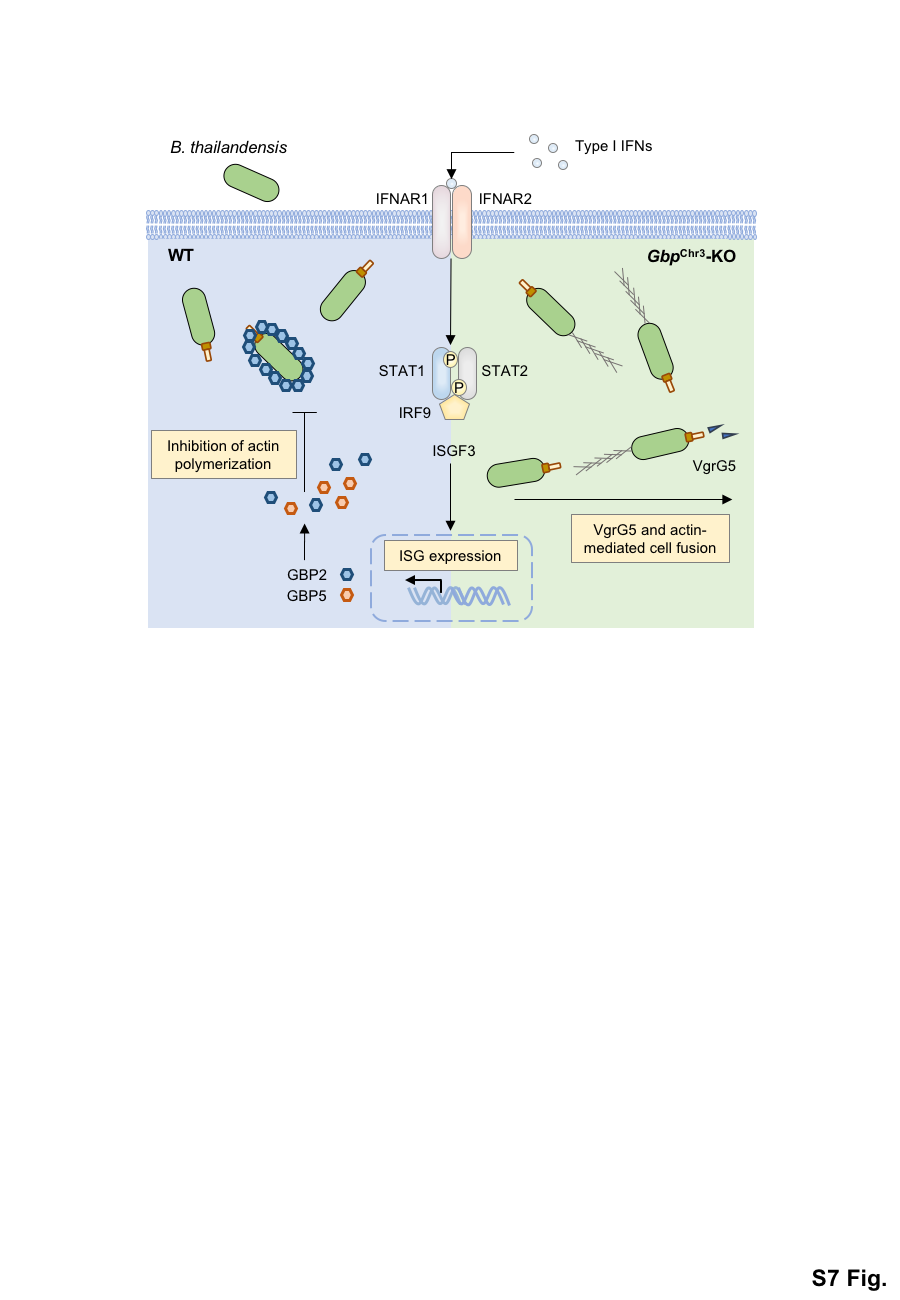

Supplement: S7 Fig — (TIFF) [file ppat.1008364.s007.tiff]
